# Evaluation of patchouli oil in the development of antibacterial nanoemulsion and nanoemulgel for periodontitis: an *in vitro* study

**DOI:** 10.3389/froh.2026.1763715

**Published:** 2026-01-22

**Authors:** Deviyanti Pratiwi, Ria Puspitawati, Dewi Fatma Suniarti, Yenny Meliana, Faisal Abnisa

**Affiliations:** 1 Doctoral Program, Faculty of Dentistry, Universitas Indonesia, Jakarta, Indonesia; 2 Department of Dental Material, Faculty of Dentistry, Universitas Trisakti, Jakarta, Indonesia; 3 Department of Oral Biology, Faculty of Dentistry, Universitas Indonesia, Jakarta, Indonesia; 4 Research Center for Molecular Chemistry, National Research and Innovation Agency, BRIN, Kawasan Puspitek, Banten, Indonesia; 5 Department of Chemical and Materials Engineering, Faculty of Engineering, King Abdulaziz University, Rabigh, Saudi Arabia

**Keywords:** antibacterial, nanoemulgel, nanoemulsion, patchouli oil, physicochemical, *pogostemon cablin* benth

## Abstract

Periodontitis is a chronic inflammatory disease driven by oral microbial dysbiosis, with *Porphyromonas gingivalis* playing as pathogenic roles and *Fusobacterium nucleatum* as a gram-negative bacterium is very relevant in the initiation and development of periodontal disease. Although local antibiotic therapy can help restore oral homeostasis, its effectiveness is often limited by bacterial resistance and poor accessibility to deep periodontal pockets. These limitations underscore the need for alternative therapies with proven antibacterial activity and biocompatibility. In this context, patchouli oil, derived from *Pogostemon cablin Benth*., offers promising antifungal, anti-inflammatory, and antibacterial properties. This study investigates the antibacterial potential of patchouli oil and its fractions, including crude, light and heavy, formulated into nanoemulsions and nanoemulgels for the adjuvant therapy of periodontitis. To achieve this, the formulation process begins by identifying which fraction meets the criteria for antibacterial efficacy. Antimicrobial characteristics are evaluated through phytochemical profiling, Fourier-transform infrared spectroscopy, and gas chromatography–mass spectrometry. The development of nanoemulsions and nanoemulgels is guided by nanomaterial formulation parameters, including physical characterization to ensure suitability for application as a mouthwash or topical paste aimed at restoring oral homeostasis. The results indicate that crude patchouli oil is a promising candidate for formulation, capable of being incorporated into nanoemulsions and nanoemulgels using surfactants and cosurfactants with appropriate hydrophilic-lipophilic balance. Furthermore, the concentration of the gelling agent significantly influences viscosity, which in turn affects the product's spreadability and retention in the oral cavity.

## 1 Introduction

Periodontitis is a prevalent chronic inflammatory disease affecting a large portion of the adult population and contributing to tooth loss and systemic complications. The condition usually begins with gingivitis, triggered by the accumulation of bacterial biofilms above and below the gum line. These biofilms disrupt the oral microbiome, leading to dysbiosis that promotes the growth of pathogenic bacteria. Among these pathogens, *Porphyromonas gingivalis* functions as a keystone species by altering the microbial environment and enhancing the virulence of other organisms such as *Fusobacterium nucleatum*. By cooperating with each other and forming biofilms that protect them from the immune system, these bacteria initiate and drive the progression of periodontal disease. Given the central role of biofilm formation in sustaining dysbiosis and tissue destruction, targeting biofilms remains a critical strategy for managing periodontitis (1–3).

Current periodontal therapies aim to suppress pathogenic bacteria associated with microbial dysbiosis, primarily through scaling and root planing (SRP) to mechanically disrupt subgingival biofilms. However, SRP is often limited in deep periodontal pockets, while adjunctive antibiotics show reduced efficacy due to rising antimicrobial resistance and biofilm-associated tolerance (4). These limitations have prompted growing interest in plant-derived bioactive compounds as adjunctive therapeutic strategies. Phytochemicals contain diverse secondary metabolites such as terpenoids, alkaloids, flavonoids, and polyphenols possess various distinct chemical structures that confer broad pharmacological activity. Importantly, these compounds exert multimodal antibiofilm effects by interfering with biofilm formation, virulence expression, and quorum sensing, thereby the microorganisms are difficult to adapt and potentially reducing the development of antimicrobial resistance (5–7).

Patchouli (*Pogostemon cablin* Benth., Lamiaceae) is a medicinal plant rich in essential oil, with patchouli alcohol (PA) as its major bioactive constituent exhibiting antimicrobial and antibiofilm activity. Patchouli oil (PO) is available in crude patchouli oil (CPO) and fractionated forms, including light-fractionated (LFPO), and heavy-fractionated (HFPO),which differ in chemical composition and PA content, thereby influencing biological activity and stability. Essential oils, including PO, have demonstrated broad-spectrum antibacterial, anti-inflammatory, and antibiofilm properties, with multi-component oils often showing superior efficacy compared with single-compound agents (8, 9). However, despite promising *in vitro* results, the clinical and preclinical translation of essential oils has been limited by their inherent volatility, poor aqueous solubility, and susceptibility to oxidative, thermal, and photodegradation. These physicochemical instabilities contribute to inconsistent antimicrobial efficacy, reduced bioavailability, and challenges in dose standardization. Accordingly, nanoformulation strategies such have been developed to improve essential-oil stability, solubility, and delivery efficiency, with multiple studies reporting enhanced antimicrobial and antibiofilm performance (5, 9, 10).

Nanoemulsions and nanoemulgels are particularly well suited for intrapocket periodontal delivery due to their nanoscale droplet size, which facilitates penetration into subgingival biofilms and close contact with the pocket epithelium. Nanoemulsions enhance the solubilization and local bioavailability of hydrophobic compounds, while their small droplet size promotes uniform distribution within the confined periodontal pocket. Incorporation of nanoemulsions into gel matrices further improves viscosity and mucoadhesion, enabling prolonged retention and controlled drug release despite continuous gingival crevicular fluid flow. These properties collectively support sustained exposure of periodontal biofilms to active agents, minimize systemic absorption, and enhance therapeutic efficacy, making nanoemulsions and nanoemulgels highly suitable platforms for localized periodontal therapy (10–12).

Despite the recognized antimicrobial potential of PO, the impact of oil fractionation on antibacterial efficacy and suitability for nano-based periodontal delivery remains unclear. Comparative evidence for CPO, LFPO and HFPO within nanoemulsion and nanoemulgel systems is currently lacking. This study therefore aims to systematically evaluate fraction-specific differences in antibacterial activity and formulation performance for periodontal therapy. Initially, CPO, LFPO, and HFPO are subjected to comprehensive physicochemical and phytochemical characterization (GC–MS and FTIR) to elucidate compositional differences. These fractions are then comparatively screened for antibacterial activity against key periodontal pathogens to identify the most effective fraction. The selected fraction is subsequently formulated into nanoemulsions and nanoemulgels using optimized surfactant, co-surfactant, and gelling systems suitable for intrapocket periodontal delivery. We hypothesize that chemical variations arising from PO fractionation will significantly influence antibacterial efficacy and that nanoemulgel formulations, particularly those incorporating 2% CPO, will demonstrate superior physical characteristics and suitability for localized periodontal application.

## 2 Materials and methods

### 2.1 Study design

**Figure 1:**
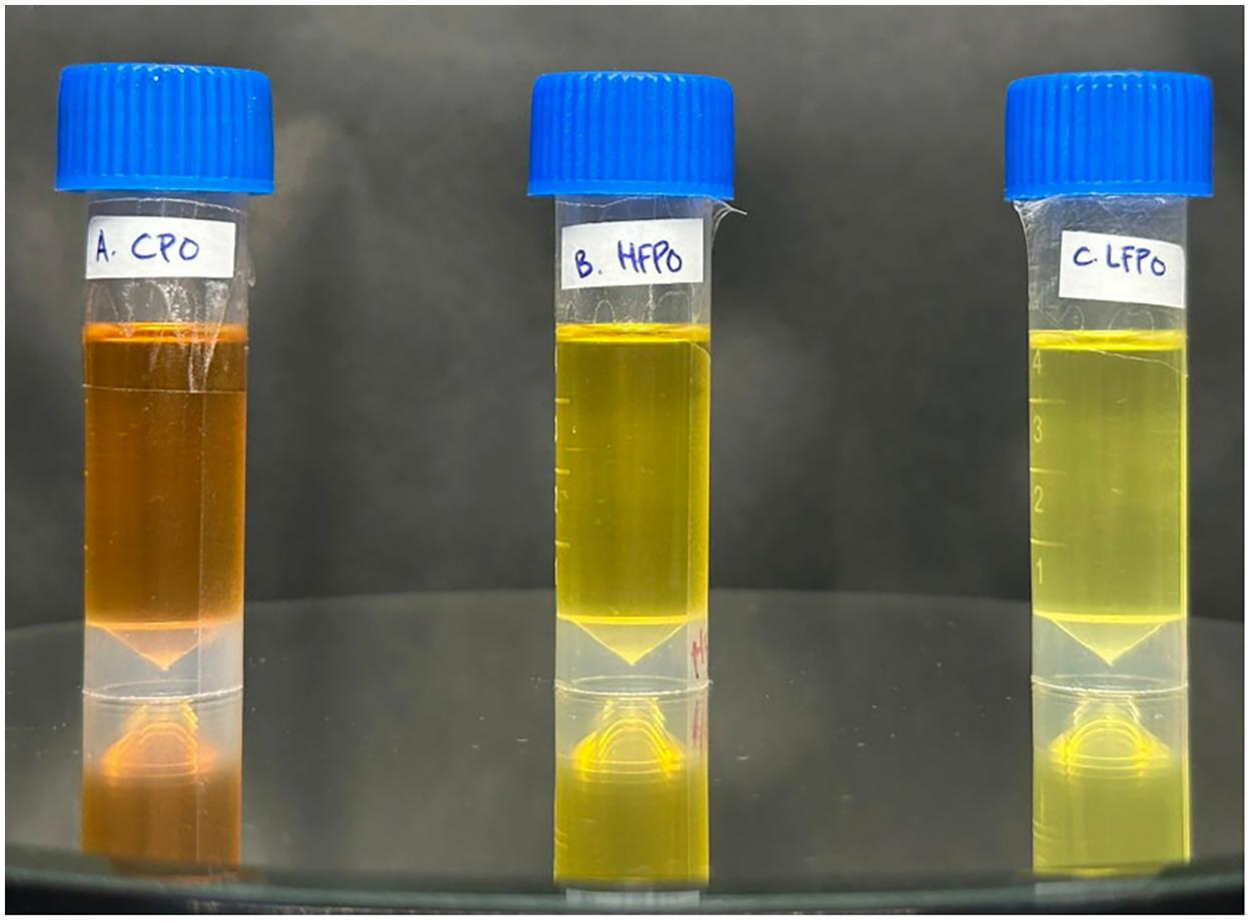
The physical appearance of the oils after extraction.

**Figure 2:**
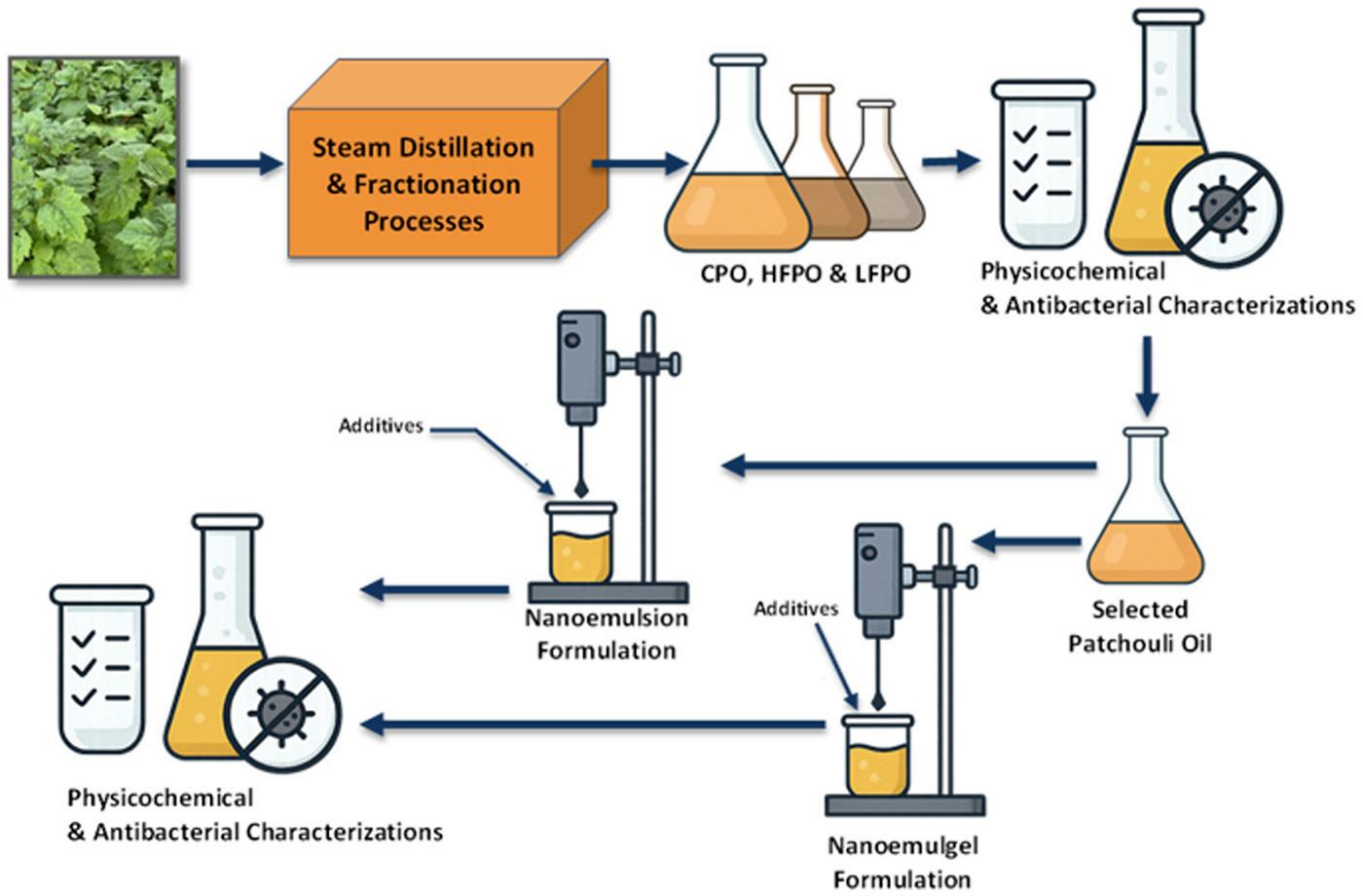
Overall workflow of patchouli oil extraction, characterization, and formulation into nanoemulsion and nanoemulgel systems.

This *in vitro* laboratory study employed a post-test only control group design to evaluate the antibacterial activity of PO and its fractions, CPO, LFPO and HFPO against *Porphyromonas gingivalis* (ATCC 33277) and *Fusobacterium nucleatum* (ATCC 25586). All three PO fractions were initially included in the screening phase to assess fraction-specific antibacterial efficacy. Based on the results of this preliminary screening, only the most effective oil fraction was selected for subsequent formulation into nanoemulsion and nanoemulgel systems to ensure optimal antibacterial performance and formulation efficiency. Chlorhexidine (CHX) and metronidazole (MTZ), widely used broad-spectrum antimicrobial agents in oral and periodontal therapy, were used as positive controls, while distilled water served as the negative control. Figure 1 shows the physical appearance of the oils after fractionation. The study further developed nanoemulsion and nanoemulgel formulations incorporating the most effective PO fraction and assessed their physicochemical and chemical characteristics. An overview of the research workflow is shown in Figure 2. The research workflow consisted of oil extraction, fractionation, characterization, antibacterial screening, and subsequent formulation of nanoemulsions and nanoemulgels based on the most promising antibacterial fraction. All procedures were carried out under controlled laboratory conditions to ensure reproducibility and analytical consistency.

### 2.2 Materials

Patchouli leaves used as the raw material for oil extraction were sourced from Aceh Province, Indonesia, a region contributing approximately 20% of the national patchouli oil supply. Indonesia is the dominant global producer of patchouli oil, accounting for an estimated 80%–90% of worldwide production (13).

Patchouli oil was obtained from *Pogostemon cablin* leaves and produced by a local manufacturer using a semi-conventional extraction approach that incorporates traditional techniques with limited technological refinement. Fresh leaves were air-dried, cut into uniform pieces, and subjected to steam distillation for 4–6 h. The resulting condensate was collected through a cooling system and separated by gravity using a separatory funnel to obtain CPO. To obtain light and heavy oil fractions, CPO was further processed using vacuum-assisted rotary evaporation under reduced pressure. Fractionation was carried out by collecting oil fractions at 65–120°C for LFPO and 120–160°C for HFPO. These fractionation steps yielded oils with distinct physicochemical and compositional profiles, which were subsequently characterized to evaluate their suitability for antibacterial assessment and formulation into nanoemulsion and nanoemulgel systems (14).

The excipients used in the formulation process included Carbopol 940, Tween 80, Span 80, propylene glycol, triethanolamine (TEA), and analytical-grade distilled water. Carbopol 940 (carboxyvinyl polymer) functions as a gelling agent that provides the structural matrix for the nanoemulgel system. Tween 80 (polysorbate 80) acts as a hydrophilic, non-ionic surfactant essential for stabilizing oil-in-water (O/W) nanoemulsions, while Span 80 (sorbitan monooleate), a lipophilic non-ionic surfactant, enhances interfacial stability by supporting emulsification of the oil phase. Propylene glycol serves as a humectant and co-solvent that improves miscibility and contributes to the uniform dispersion of the oil droplets. TEA is employed as a neutralizing and pH-adjusting agent to activate Carbopol, enabling proper gel formation. Distilled water of analytical grade is used as the aqueous phase for all formulations. Together, these materials enable the preparation of stable nanoemulsion and nanoemulgel systems suitable for evaluating the antibacterial potential of patchouli oil.

### 2.3 Methods

#### 2.3.1 Preparation of nanoemulsions and nanoemulgels

The oil fraction exhibiting the highest antibacterial activity was selected for the preparation of nanoemulsions and nanoemulgels Nanoemulsions were formulated by incorporating patchouli oil at concentrations of 2% and 5% (w/v) into an oil-in-water system. Tween 80 (HLB 15) and Span 80 (HLB 4.3) were used as the hydrophilic and lipophilic surfactants, respectively. The surfactant blend was designed to achieve a target hydrophilic–lipophilic balance (HLB) suitable for oil-in-water emulsification (HLB 8–18), calculated using the weighted-average equation:

$$HLB=\frac{\left(Wa\times HLBa)+(Wb\;\times HLBb\right)}{Wa+Wb}$$

where Wa and Wb represent the weight fractions of each surfactant relative to the total surfactant concentration. Propylene glycol was incorporated as a co-surfactant and humectant to improve oil dispersion and system stability. Distilled water was added to adjust the final formulation volume to 100 mL (10).

The oil and aqueous phases were first combined to form a coarse emulsion, followed by high-speed homogenization at 6,000 rpm for 15 min. Triethanolamine was added in minimal amounts to adjust pH and enhance nanoemulsion stability. Two primary nanoemulsion formulations (F1–F2), differing in oil concentration and surfactant ratios, were prepared (Table 1).

**Table 1:** Formulations of nanoemulsions and nanoemulgels with varying oil and carbopol concentrations.

Ingredients (%w/w)	F1	F2	F3	F4	F5	F6	F7	F8
Essential oil (CPO)	2	5	2	2	2	5	5	5
Tween 80	12,5	15	6	6	6	10	10	10
Span 80	7,5	9	2	2	2	4	4	4
Propylen Glikol	10	10	5	5	5	5	5	5
Carbopol 940			0,2	0,3	0,4	0,2	0,3	0,4
Triethanolamine	0,025	0,025	0,5	0,5	0,5	0,3	0,3	0,3
Distilled water	q.s.	q.s.	q.s.	q.s.	q.s.	q.s.	q.s.	q.s.

Nanoemulgels were prepared by dispersing Carbopol® 940 in preserved water and allowing complete hydration for 24 h at room temperature. Selected nanoemulsions (2% and 5% oil) were gradually incorporated into the hydrated Carbopol matrix under continuous stirring. TEA was subsequently added to neutralize the polymer and induce gel formation. Final homogenization was performed at 10,000–12,000 rpm for 10 min to ensure uniform droplet distribution.

High-speed homogenization was carried out using an Ultra-Turrax homogenizer (IKA T25 digital, Germany). Temperature during all preparation steps was monitored and maintained below 40°C to preserve thermosensitive constituents of patchouli oil. Nanoemulgels were formulated using three Carbopol concentrations (0.2%, 0.3%, and 0.4%) at both oil levels, resulting in six nanoemulgel formulations (F3–F8). Complete compositional details of all nanoemulsion and nanoemulgel formulations (F1–F8) are provided in Table 1.

The selection of 2% and 5% oil concentrations was guided by previous reports indicating that patchouli oil levels below 10% provide acceptable physicochemical stability and preserved antibacterial activity, while higher concentrations may adversely affect formulation stability, handling properties, and safety (15). The dosage form does not alter the intrinsic antibacterial activity of patchouli oil, as both emulsions and gels remain effective at inhibiting bacterial growth across various concentrations. Increasing the oil concentration typically enhances antibacterial inhibition, however, it may also affect the physical characteristics of the formulation, such as viscosity, stability, and ease of application (16). The associated challenges of EO, such as limited bioavailability, stability, and potential toxicity at higher concentrations (17).

### 2.4 Characterization of patchouli oil and formulations

#### 2.4.1 Physical characterization

Qualitative phytochemical screening of CPO, LFPO and HFPO was conducted using standard colorimetric and precipitation assays to identify major secondary metabolites, including alkaloids (Dragendorff and Bouchardat), saponins (foam test), tannins (FeCl₃), phenolics (NaOH), flavonoids (H₂SO₄), glycosides (Keller–Killiani), and triterpenoids/steroids (Liebermann–Burchard). All assays were performed at room temperature (25 ± 2°C) and used for preliminary compositional profiling to support fraction selection.

Organoleptic properties (color, clarity, homogeneity, and phase separation) of all nanoemulsion and nanoemulgel formulations were evaluated by visual inspection during storage. The pH of each formulation was measured in triplicate at 25°C using a calibrated benchtop pH meter (buffers pH 4.0, 7.0, and 10.0), with the electrode immersed directly into the formulation without dilution until a stable reading was obtained.

Viscosity measurements were performed using a Brookfield DV-II+ rotational viscometer (AMETEK Brookfield, Middleboro, MA, USA) following the manufacturer's guidelines. Measurements were conducted at 25 ± 1°C, with spindle type and rotational speed selected to maintain torque values within the recommended range of 10%–90%. Spindle 61 was used for low-viscosity nanoemulsions, while spindle 64 was applied for high-viscosity nanoemulgel formulations. Each sample was measured in triplicate with a 60 s reading time, and results were expressed as mean ± standard deviation (SD).

Particle size distribution, polydispersity index (PDI), and zeta potential were determined by dynamic light scattering using a Horiba SZ-100 analyzer at a scattering angle of 90° and a temperature of 25°C. Samples were appropriately diluted to minimize multiple scattering effects. Zeta potential measurements were performed using a Malvern Zetasizer under electrophoretic light scattering mode. Spreadability of nanoemulgel formulations was assessed using a slip-and-drag method by placing 100 g weight on the upper glass slide for 1–2 min, and the spread diameter was measured in two perpendicular directions and averaged.

#### 2.4.2 Chemical and antibacterial characterization

The chemical composition of PO and its fractions was analyzed using gas chromatography–mass spectrometry (GC–MS) (Shimadzu GC-2010 Plus) equipped with a TG-5MS capillary column (30 m × 0.25 mm i.d., 0.25 µm film thickness). The oven temperature program was set at 60°C (held for 4 min), increased to 150°C (held for 4 min), and then ramped to 250°C. Helium was used as the carrier gas at a constant flow rate of 1.0 mL/min. Mass spectra were acquired under electron-impact ionization at 70 eV, with a scan range of m/z 40–500. Compound identification was performed by comparison with NIST mass spectral libraries. Functional group characterization was conducted using attenuated total reflectance–Fourier transform infrared spectroscopy (ATR-FTIR). Spectra were recorded in the range of 4,000–500 cm^−1^ at a spectral resolution of 4 cm^−1^.

Antibacterial activity was evaluated using the agar well diffusion method in accordance with commonly accepted microbiological procedures. Agar medium was poured to a uniform depth of approximately 4 mm, and wells with a diameter of 6 mm were aseptically punched into the solidified agar. Bacterial suspensions of *Porphyromonas gingivalis* and *Fusobacterium nucleatum* were adjusted to 0.5 McFarland turbidity (≈1 × 10^7 ^CFU/mL) and evenly inoculated onto the agar surface. Each well was loaded with 25 µL of the test sample or control. Plates were incubated at 37°C for 24 h under strict anaerobic conditions using an anaerobic jar with a gas-generating system. Antibacterial activity was assessed by measuring the diameter of the inhibition zones (mm).

The data from all experiments were presented as mean ± SD. All the experiments were done in two independent experiments with at least three replicates in each experiment (*n* = 6 per group), unless indicated within figures. Data normality was assessed by using either QQ plots or the Shapiro–Wilk test. If the data were normally distributed, significance analysis was done using two-way ANOVA, followed by Tukey's multiple comparisons test. Meanwhile, if the data were not normally distribute, significance analysis was done using Kruskal Wallis, followed by Dunn's multiple comparisons test. The detail statistical analysis was indicated in the figure caption. For all the analyses, *p*-value < 0.05 was considered significantly different. All the statistical analyses were performed in GraphPad Prism software (version 10.5.0 for Mac OS; GraphPad Software, Boston, MA, USA).

## 3 Results

### 3.1 Phytochemical screening

Qualitative phytochemical screening demonstrated that CPO, LFPO and HFPO, all tested positive for alkaloids, saponins, tannins, phenolics, flavonoids, glycosides, and triterpenoids. Steroid compounds were not detected in any of the samples. The screening results indicate the presence of similar classes of secondary metabolites across all patchouli oil types, providing qualitative confirmation of their phytochemical profiles.

### 3.2 Quantitative chemical and phytochemical composition

Quantitative analysis revealed distinct differences in the chemical and phytochemical composition among CPO and its fractionated products, LFPO and HFPO (Table 2). HFPO exhibited the highest patchouli alcohol content (56.72% by GC), compared with 30.96% in CPO and 21.49% in LFPO. Minor phytochemical constituents, including tannins, saponins, and flavonoids, were present at low concentrations across all samples, with tannin levels ranging from 0.04%–0.06%, saponins from 0.09%–0.10%, and flavonoids (expressed as quercetin equivalents) from 0.11%–0.17%, showing only modest variation among oil types. Antioxidant activity, expressed as IC_50_ values, also differed among samples, with LFPO exhibiting the highest IC_50_ value, followed by HFPO and CPO. These results provide an objective, quantitative comparison of the compositional differences among CPO and its fractionated products, while phytochemical screening assays served as qualitative confirmation.

**Table 2:** Quantitative chemical and phytochemical composition of patchouli oil (CPO, LFPO, HFPO).

Type of testing	Results			Methods
	CPO	LFPO	HFPO	
Patchouly Alcohol (%GC)	30.96	21.49	56.72	GC
Tannin (%)	0.04	0.06	0.04	Spectrophotometer
Saponin (%)	0.09	0.10	0.09	TLC Scanner
Flavonoids as Querstein (%)	0.11	0.17	0.17	Spectrophotometer
Antioxidant Activity IC 50% (%)	0.42	0.79	0.59	Spectrophotometer

### 3.3 Physical characteristics

#### 3.3.1 Characteristics of patchouli oil fractions

As shown in Figure 1 and Table 3, CPO, LFPO and HFPO exhibited distinct physical characteristics. CPO appeared dark brown to deep reddish brown with a strong and slightly pungent aroma, whereas HFPO was clear brownish-yellow with a smoother patchouli aroma. LFPO showed the highest clarity, appearing light yellow to almost clear, with a lighter and fresher aroma. Quantitative measurements revealed differences in viscosity and pH among the oils. HFPO exhibited the highest viscosity (61.92 ± 1.2 cP), followed by CPO (30.12 ± 1.2 cP), while LFPO showed the lowest viscosity (25.56 ± 1.2 cP), corresponding to its thinner and more freely flowing appearance. The pH values of the oils ranged from 5.04 (HFPO) to 6.64 (LFPO), with CPO showing an intermediate pH of 5.43.

**Table 3:** Characteristics of patchouli oil (CPO, LFPO, HFPO). The three types of oil have different physical characteristic.

Characteristic	CPO	LFPO	HFPO
Color	Dark brown/deep reddish brown	Light yellow to almost clear	Clear brownish yellow
Odor/Aroma	Strong, distinctive patchouli, slightly pungent	Light, softer, subtle patchouli aroma with freshness	Smoother, still strong, soft patchouli aroma
Clarity	Cloudy	Very clear	Clearer than CPO
Visual appearance and viscosity (cP)	Thick	Thinner, flows easily	Thick
	30,12 ± 1,2	25,56 ± 1,2	61,92 ± 1,2
pH	5,43	6,64	5,04

#### 3.3.2 Physicochemical properties of nanoemulsion and nanoemulgel formulations

The physicochemical properties of the nanoemulsion and nanoemulgel formulations are summarized in Table 4. All formulations (F1–F8) exhibited good consistency and excellent homogeneity, with colors ranging from white to yellowish-white. The pH of all formulations ranged from 6.10 to 6.57, falling within a range suitable for intraoral application. Nanoemulsion formulations (F1 and F2) and nanoemulgel F6 exhibited low viscosities (13.4–928 cP), whereas the remaining nanoemulgel formulations showed substantially higher viscosities (4,200–20,890 cP) depending on Carbopol concentration. Formulations containing 0.4% Carbopol (F5 and F8) exhibited the highest viscosities, with values of 14,150 and 20,890 cP, respectively. Spreadability values decreased with increasing viscosity, ranging from 10.0 ± 0.2 g·cm/s in low-viscosity formulations (F1) to 5.1 ± 0.6 g·cm/s in higher-viscosity nanoemulgels (F8). These results demonstrate that modulation of formulation composition effectively controlled viscosity and spreadability, resulting in formulations with distinct handling characteristics.

**Table 4:** Physicochemical properties of nanoemulsion and nanoemulgel.

Parameters	F1	F2	F3	F4	F5	F6	F7	F8
Color	White	yellowish white	white	white	white	yellowish white	yellowish white	yellowish white
Consistency	Good	Good	Good	Good	Good	Good	Good	Good
Homogeneity	Excellent	Excellent	Excellent	Excellent	Excellent	Excellent	Excellent	Excellent
pH	6,39	6,34	6,17	6,31	6,57	6,10	6,28	6,25
Viscosity (cP)	13,4	36,52	4,200	13,080	14,150	928	5,690	20,890
Spreadability (g·cm/s)	10 ± 0,2	8,12 ± 0,1	8 ± 0,1	6,8 ± 0,1	5,9 ± 0,1	7 ± 0,1	5,6 ± 0,6	5,1 ± 0,6

Particle size analysis showed that all nanoemulsion and nanoemulgel formulations (F1–F8) exhibited nanoscale dimensions at room temperature, with mean particle sizes ranging from 100.0 ± 2.2 nm to 152.7 ± 2.2 nm (Table 5). Among the formulations tested, F5 exhibited the smallest particle size (100.0 ± 2.2 nm), whereas the largest particles were observed in F7 (152.7 ± 2.2 nm). Polydispersity index (PDI) values ranged from 0.46 to 0.82, indicating moderate size distribution across formulations. All formulations exhibited high-magnitude negative zeta potential values, ranging from −71.5 ± 0.4 mV to −95.4 ± 0.5 mV, suggesting good electrostatic stability. Collectively, these results indicate that all formulations possessed nanoscale particle sizes with acceptable dispersion characteristics and high colloidal stability.

**Table 5:** Physical characteristics nanoemulsion and nanoemulgel at room temperature.

Sample	Particle size (nm)	PDI	Zeta potensial (mV)
F1	130,0 ± 2,0	0,46	(−) 95,4 ± 0,5
F2	144,5 ± 2,1	0,75	(−) 80,5 ± 0,3
F3	127,6 ± 2,2	0,80	(−) 81,3 ± 0,6
F4	122,7 ± 2,1	0,64	(−) 84,4 ± 0,4
F5	100,0 ± 2,2	0,50	(−) 85,2 ± 0,4
F6	152,4 ± 2,1	0,72	(−) 80,6 ± 0,6
F7	152,7 ± 2,2	0,82	(−) 93,3 ± 0,1
F8	147,6 ± 2,1	0,70	(−) 71,5 ± 0,4

### 3.4 Chemical characterization

#### 3.4.1 GCMS

GC–MS analysis showed that CPO, LFPO and HFPO shared a similar qualitative composition, consisting mainly of sesquiterpene hydrocarbons and oxygenated sesquiterpenes (Table 6). Major constituents detected in all samples included patchouli alcohol, globulol, α-guaiene, seychellene, aciphyllene, and azulene. Quantitative comparison based on relative peak area percentages (%) revealed clear differences among the oils. HFPO was enriched in oxygenated sesquiterpenes, particularly patchouli alcohol (49,16%) and globulol (6,76%), compared with CPO (31,42% and 2,76%) and LFPO (21.80% and 1,99%). In contrast, LFPO showed higher proportions of sesquiterpene hydrocarbons such as α-guaiene and seychellene, whereas CPO exhibited a more evenly distributed composition without dominance of a single constituent.

**Table 6:** GC–MS–identified major constituents of patchouli oil, including CPO, LFPO and HFPO showing retention times (min) and relative peak area percentages (%).

Components	CPO		LFPO		HFPO		Description
	Retention time (min)	Peak area (%)	Retention time (min)	Peak area (%)	Retention time (min)	Peak area (%)	
*α*-guaiene	20,938	15,20	20,959	16,63	20,928	5,54	Sesquiterpene hydrocarbon
Secychellean	21,064	7,49	21,081	10,74	21,061	3,32	Sesquiterpene hydrocarbon
Aciphyllene	22,445	3,88	22,448	5,34	22,442	3,80	Sesquiterpene hydrocarbon
Azulene	22,621	21,02	22,645	22,08	22,629	19,25	Sesquiterpene hydrocarbon
Globulol	26,125	2,76	26,121	1,99	26,135	6,76	Oxygenated sesquiterpene
Patchouli alcohol	26,264	31,42	26,285	21,80	26,315	49,16	Oxygenated sesquiterpene

#### 3.4.2 FTIR

**Figure 3:**
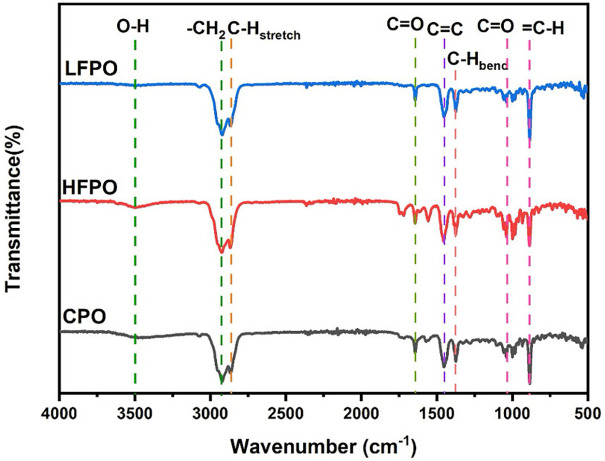
FTIR spectra of CPO, LFPO, and HFPO showing similar absorption patterns and comparable functional groups; compound-level identification was determined by GC–MS.

FTIR spectra of CPO, LFPO, and HFPO exhibited highly similar absorption patterns, indicating comparable functional groups across all samples (Figure 3). Broad bands at 3,300–3,500 cm^−1^ were assigned to O–H stretching vibrations, while strong absorptions in the 2,850–2,950 cm^−1^ region corresponded to aliphatic C–H stretching. Additional characteristic bands in the fingerprint region (1,700–1,000 cm^−1^) were associated with C = O and C = C stretching, as well as C–H bending vibrations. FTIR analysis was therefore used to confirm functional group similarity among the oils, whereas compound-level identification and relative abundance determinations were derived exclusively from GC–MS analysis.

### 3.5 Antibacterial activity

#### 3.5.1 Antibacterial activity of patchouli oil fractions

Antibacterial activity of patchouli oil fractions was measured by the diameter of zone inhibition. According to the results presented in Table 1, the antibacterial activity of patchouli oil was significantly different on *P. gingivalis* and *F. nucleatum* (*p-value* < *0.05*)*.* Specifically, the antibacterial activity showed higher efficiency on *F. nucleatum,* compared to *P. gingivalis.* These results suggested that oral microorganism has different susceptibility against antimicrobial agents, including patchouli oil.

**Figure 4:**
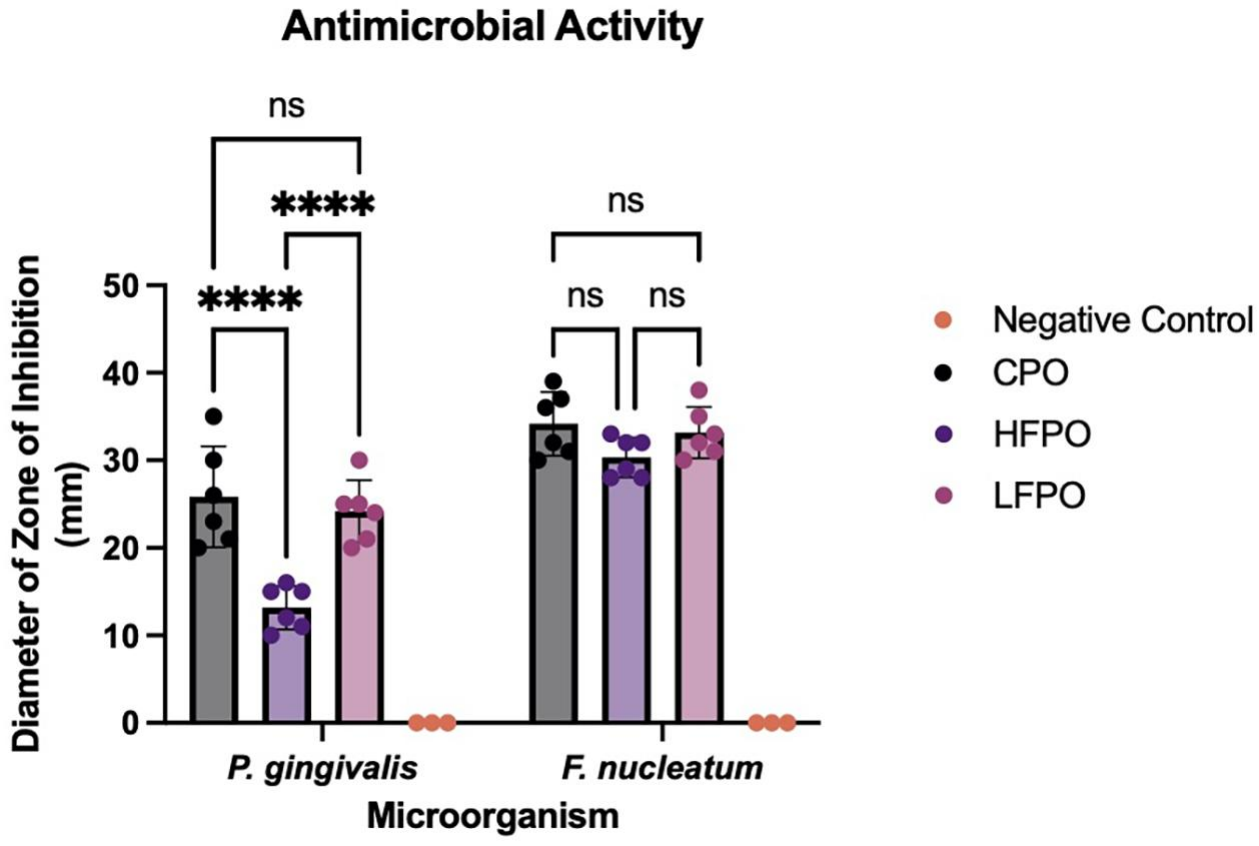
Antibacterial activity of CPO, LFPO and HFPO against *P. gingivalis* (left) and *F. nucleatum* (right). Data are shown as mean ± SD (*n* = 6). Statistical analysis was conducted using two-way ANOVA followed by Tukey's multiple comparisons test. ns: not significant, **** *p-value* < 0.0001.

Moreover, the patchouli oil fractions showed a significantly different antimicrobial activity. This effect can be seen clearly in *P. gingivalis* group. According to the results presented in Figure 4, it was shown that CPO and LFPO had better antibacterial activity compared to HFPO. In contrast, there was no significant difference on the antibacterial activity of patchouli oil fractions in *F. nucleatum,* suggesting that all the fractions showed similar antibacterial efficiency against *F. nucleatum*.

#### 3.5.2 Antibacterial activity of nanoemulsion and nanoemulgel formulations

To improve the oil fractions stability, we formulated the oil fractions in a nanoemulsion and nanoemulgel form. Next, we tested the antibacterial activity of nanoemulsion and nanoemulgel of patchouli oil fractions with the similar methods as in previous section. Since we could not detect any significant differences between CHX and MTZ, we compared the antimicrobial activity of the oil groups to CHX. Therefore, oil groups with non-significant *p-value* showed comparable antimicrobial activity with CHX, the positive control.

**Figure 5:**
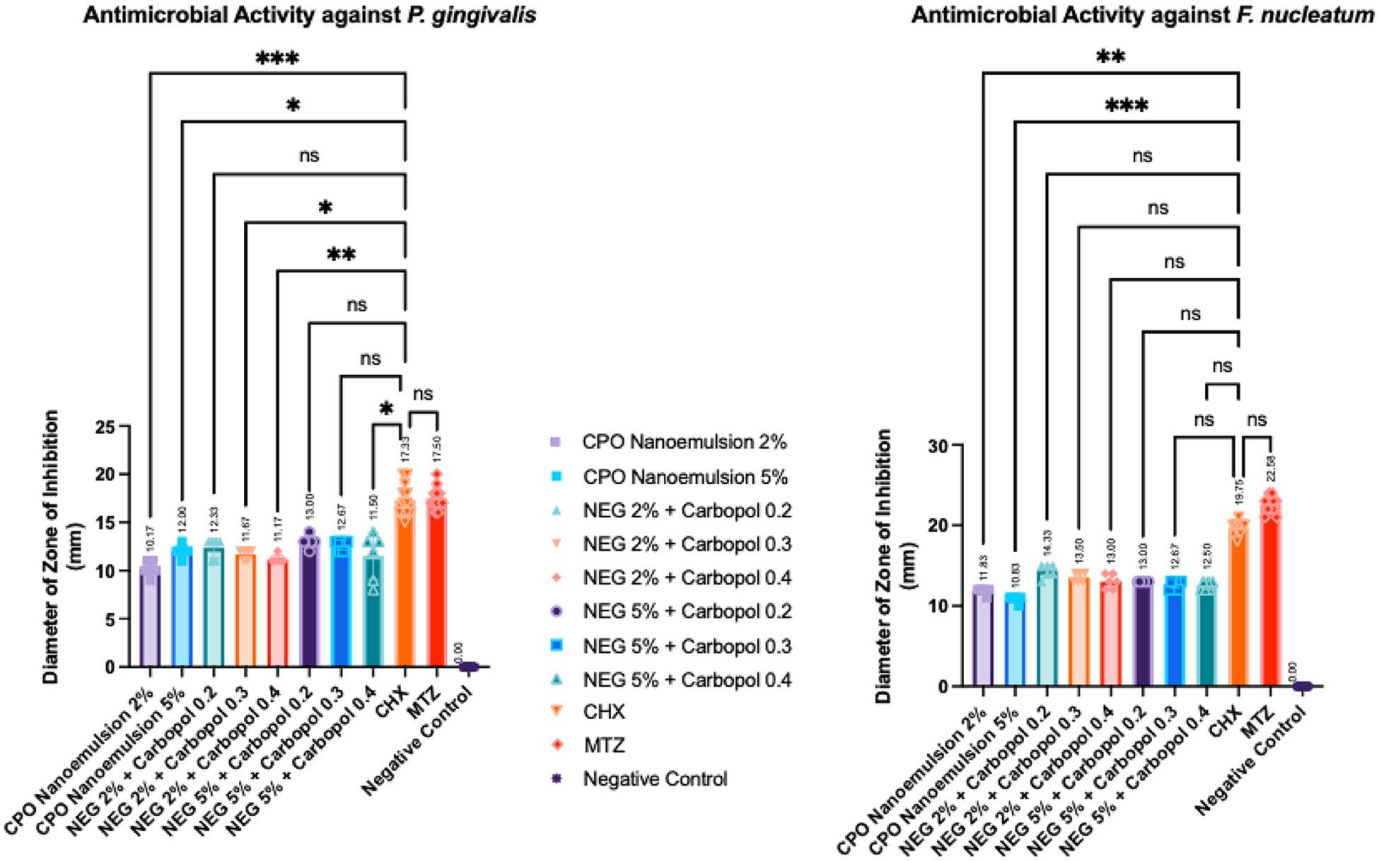
Antibacterial activity of nanoemulsion and nanoemulgel formulations against *P. gingivalis* **(a)** and *F. nucleatum* **(b)**. Data are shown as mean ± SD (*n* = 6 for each nanoemulsion and nanoemulgel formulation; *n* = 12 for chlorhexidine (CHX), metronidazole (MTZ), and negative control). Statistical analysis was performed using the Kruskal–Wallis test followed by Dunn's multiple comparisons test.

For *p. gingivalis,* non-significant differences were detected in NEG 2% + Carbopol 0.2, NEG 5% + Carbopol 0.2, and NEG 5% + Carbopol 0.3 groups. Meanwhile, for *F. nucleatum*, nonsignificant differences were detected in Carbopol-supplemented NEG 2% and NEG 5%, as shown in Figure 5. In each oral microorganisms, the highest average diameter of zone inhibition was detected when the nanoemulgel was supplemented with Carbopol 0.2%. Therefore, it is suggested that formulating patchouli oil in a nanoemulgel and supplemented with 0.2 Carbopol can enhance the antibacterial activity against *P. gingivalis* and *F. nucleatum*.

## 4 Discussion

### 4.1 Effect of patchouli oil fractionation as an antibacterial agent

Essential oils are complex mixtures of volatile bioactive constituents whose composition is influenced by botanical origin, environmental conditions, and processing techniques such as extraction and fractionation (17). Owing to this multicomponent nature, essential oils have been widely reported to exhibit *in vitro* antibacterial activity against periodontal pathogens through mechanisms that include membrane disruption, interference with enzymatic pathways, and modulation of biofilm formation (11, 18).

In the present study, fractionation of patchouli oil led to a redistribution of its constituents, as demonstrated by GC–MS analysis, while FTIR spectra confirmed the absence of new functional groups. These results indicate that fractionation alters the relative abundance of existing compounds rather than generating new chemical entities, consistent with volatility- and boiling-point-based separation in which lower-boiling components accumulate in LFPO and higher-boiling components in HFPO. Chemical profiling further revealed distinct compositional differences between CPO and its fractions, with PA remaining the predominant constituent and showing greater enrichment in HFPO than in LFPO (18–21).

Despite this higher enrichment of PA in HFPO, CPO produced larger inhibition zones against *Porphyromonas gingivalis* and *Fusobacterium nucleatum* in agar diffusion assays. These observations suggest that antibacterial activity is not dictated by a single dominant compound but is more plausibly mediated by additive or synergistic interactions among multiple phytochemicals present in the unfractionated oil. Previous studies similarly indicate that sesquiterpenes, phenolics, and other minor constituents in *Pogostemon cablin* act together to disrupt bacterial membranes, impair metabolic activity, and attenuate biofilm formation (8, 9, 21). Consistent with the present findings, other investigations have reported that patchouli oil demonstrates medium antibacterial activity (inhibition zones ∼11–12 mm), lower than positive-control antibiotics (>18 mm) yet clearly higher than negative controls (0 mm) (14). These comparisons reinforce that while patchouli oil exhibits measurable antibacterial potential, inhibition-zone data remain qualitative indicators (11, 14).

In the context of this work, the discussed mechanisms are intended to support interpretation, particularly in guiding the selection of the most appropriate oil type for nanoemulsion and nanoemulgel formulations. Overall, the findings indicate that fractionation modifies both the chemical composition and *in vitro* antibacterial inhibition profile of patchouli oil, whereas the broader compositional complexity of CPO may confer advantages through multicomponent interactions.

### 4.2 Influence of formulation on the characteristics of nanoemulsion and nanoemulgel as antibacterial agents for periodontitis

The antibacterial performance of natural compounds is strongly determined by their physicochemical properties, including stability, solubility, and bioavailability (17, 22). Consequently, formulation becomes a key step for optimizing antibacterial effectiveness, particularly through the development of nano-based delivery systems. In this context, each component and processing condition, such as oil concentration, surfactant level, and homogenization technique, directly influences particle size, polydispersity index (PDI), zeta potential, and ultimately the biological performance of the formulation (5, 10, 11, 14).

The present findings demonstrate that increasing the oil-phase concentration tends to enlarge particle size and increase PDI when the surfactant content is insufficient to coat the entire droplet surface, thereby increasing interfacial tension and promoting coalescence. Conversely, higher stirring speed, sonication, and surfactant concentration produced smaller droplets (approximately 200–500 nm) with narrower size distributions (23). These results are consistent with previous reports showing that high-energy homogenization reduces droplet size through intense shear and cavitation, generating nanosized systems with larger surface area and improved antibacterial interaction potential (11, 14).

All eight formulations developed in this study fulfilled the criteria of stable nanoemulsion/nanoemulgel systems. Formulations containing 2% patchouli oil consistently exhibited smaller particle sizes, narrower distributions, and higher absolute zeta potentials than 5% formulations, indicating greater emulsification efficiency and improved physical stability at lower oil concentrations. Increasing oil concentration significantly increased particle size in both nanoemulsions (F2 > F1) and nanoemulgels (F6–F8 > F3–F5), consistent with surfactant limitation at higher dispersed-phase volumes.

Formulation success was also influenced by the appropriateness of the hydrophilic–lipophilic balance (HLB) and the critical packing ratio (CPP). The use of Tween 80 and Span 80 matched the HLB range of patchouli oil (≈10–12), enabling the formation of stable interfacial films with minimal aggregation risk. In nanoemulgels, incorporation of nanoemulsions into a Carbopol-based polymeric matrix allowed control of viscosity, spreadability, and retention on gingival tissues, parameters essential for topical periodontal application (24–26).

The Carbopol concentrations selected in this study (0.5%–2%) were consistent with established formulation guidelines, where increasing concentration increases gel viscosity (27). Neutralization with TEA was required to stabilize the polymer network and generate an optimal gel structure. In accordance with our results, viscosity was inversely related to spreadability, which directly influences the diffusion of active ingredients across the oral mucosa. Spreadability was additionally affected by formulation composition, particularly the balance between gelling agents and humectants that maintain structural integrity and moisture (24, 25, 27).

Beyond particle characteristics, the pH and viscosity profiles of the formulations were within biocompatible ranges. The observed pH values (6.10–6.57) are appropriate for oral tissues and do not disrupt the salivary environment. Low-viscosity nanoemulsions favored extensive distribution in the oral cavity, whereas higher-viscosity nanoemulgels provided superior adhesion and residence time in periodontal pockets (28).

### 4.3 Suggestion

Future research should broaden antibacterial assessment to polymicrobial periodontal biofilm models that more accurately reflect biofilm maturity, interspecies interactions, and ecological complexity. The incorporation of quantitative antimicrobial methodologies (such as MIC and MBC determinations), together with biofilm disruption analyses and *in vivo* periodontal models, is strongly recommended to elucidate potential synergistic interactions among PO constituents and to explain the superior antibacterial performance of crude oil relative to its fractionated forms. Such studies should also aim to confirm these synergistic effects and to comprehensively evaluate the safety, stability, biocompatibility, and therapeutic relevance of patchouli-oil–based nanoemulsion and nanoemulgel formulations for periodontal applications.

## 5 Conclusion

This study demonstrates that fractionation alters the chemical composition of PO and modulates its *in vitro* antibacterial activity against *P. gingivalis* and *F. nucleatum*. Although HFPO contained higher levels of PA, CPO produced larger inhibition zones, indicating that antibacterial activity is likely driven by multicomponent interactions rather than a single dominant constituent. CPO based nanoemulsion and nanoemulgel formulations exhibited favorable physicochemical characteristics, supporting their potential as topical adjunctive antibacterials for periodontitis. Importantly, the present findings highlight that the development of CPO nanoformulations should carefully consider HLB matched emulsifier systems and appropriate gelling agent concentrations, as these parameters critically influence particle size, stability, viscosity, and overall clinical usability.

## Data Availability

The original contributions presented in the study are included in the article/Supplementary Material, further inquiries can be directed to the corresponding author.
