# Supplementary material for: Evaluation of patchouli oil in the development of antibacterial nanoemulsion and nanoemulgel for periodontitis: an in vitro study
Source: Front Oral Health. 2026 Jan 22;7:1763715. doi: 10.3389/froh.2026.1763715 (PMC12872485; doi:10.3389/froh.2026.1763715)
Supplement: Supplementary file 1 [file Datasheet1.pdf]

frontiers

Supplementary Tables

Supplementary Table S1. Two-way ANOVA results for antibacterial activity

**Source of variation**	**SS (type III)**	**DF**	**MS**	**F (DFn, DFd)**	***p-value***
Interaction	315.2	3	147.6	$F(3, 40) = 14.93$	$P < 0.0001$
Microorganism	714.2	1	892.7	$F(1, 40) = 90.28$	$P < 0.0001$
Oil Fraction	4166	3	2313	$F(3, 40) = 233.9$	$P < 0.0001$
Residual	395.5	40	9.888		

Supplementary Table S2. Tukey’s post hoc analysis for antibacterial activity

Tukey's multiple comparisons test	Summary	Adjusted P Value
P. gingivalis		
CPO vs. HFPO	****	<0.0001
CPO vs. LFPO	ns	0.7954
CPO vs. Negative Control	****	<0.0001
HFPO vs. LFPO	****	<0.0001
HFPO vs. Negative Control	****	<0.0001
LFPO vs. Negative Control	****	<0.0001
F. nucleatum		
CPO vs. HFPO	ns	0.1669
CPO vs. LFPO	ns	0.9458
CPO vs. Negative Control	****	<0.0001
HFPO vs. LFPO	ns	0.4121
HFPO vs. Negative Control	****	<0.0001
LFPO vs. Negative Control	****	<0.0001

Supplementary Table S3. Non-parametric analysis of antibacterial activity (Nanoemulsion and Nanoemulgel)

Kruskal-Wallis test for *P. gingivalis*

P value	<0.0001
Exact or approximate P value?	Approximate
P value summary	****
Do the medians vary signif. (P < 0.05)?	Yes
Number of groups	11
Kruskal-Wallis statistic	73.94

Kruskal-Wallis test for *F. nucleatum*

P value	<0.0001
Exact or approximate P value?	Approximate
P value summary	****
Do the medians vary signif. (P < 0.05)?	Yes
Number of groups	11
Kruskal-Wallis statistics	79.02

Supplementary Table S4. Dunn’s post hoc test comparing formulations with positive controls (*P. gingivalis*)

Dunn's multiple comparisons test	Summary	Adjusted P Value
CHX vs. CPO Nanoemulsion 2%	***	0.0001
CHX vs. CPO Nanoemulsion 5%	*	0.0430
CHX vs. NEG 2% + Carbopol 0.2	ns	0.1432
CHX vs. NEG 2% + Carbopol 0.3	*	0.0128
CHX vs. NEG 2% + Carbopol 0.4	**	0.0019
CHX vs. NEG 5% + Carbopol 0.2	ns	0.7271
CHX vs. NEG 5% + Carbopol 0.3	ns	0.3532
CHX vs. NEG 5% + Carbopol 0.4	*	0.0478
CHX vs. MTZ	ns	>0.9999
CHX vs. Negative Control	****	<0.0001

Supplementary Table S5. Dunn's post hoc test comparing formulations with positive controls (*F.nucleatum*)

Dunn's multiple comparisons test	Summary	Adjusted P Value
CHX vs. CPO Nanoemulsion 2%	**	0.0042
CHX vs. CPO Nanoemulsion 5%	***	0.0003
CHX vs. NEG 2% + Carbopol 0.2	ns	>0.9999
CHX vs. NEG 2% + Carbopol 0.3	ns	>0.9999
CHX vs. NEG 2% + Carbopol 0.4	ns	0.2872
CHX vs. NEG 5% + Carbopol 0.2	ns	0.3415
CHX vs. NEG 5% + Carbopol 0.3	ns	0.1089
CHX vs. NEG 5% + Carbopol 0.4	ns	0.0578
CHX vs. MTZ	ns	>0.9999
CHX vs. Negative Control	****	<0.0001
